# Identification of novel biomarkers in septic cardiomyopathy *via* integrated bioinformatics analysis and experimental validation

**DOI:** 10.3389/fgene.2022.929293

**Published:** 2022-07-25

**Authors:** Feng Lu, Feng Hu, Baiquan Qiu, Hongpeng Zou, Jianjun Xu

**Affiliations:** ^1^ Department of Cardiothoracic Surgery, The Second Affiliated Hospital of Nanchang University, Nanchang, China; ^2^ Department of Cardiovascular Medicine, The Second Affiliated Hospital of Nanchang University, Nanchang, China

**Keywords:** septic cardiomyopathy, sepsis, biomarkers, integrated bioinformatics analysis, differentially expressed genes

## Abstract

**Purpose:** Septic cardiomyopathy (SCM) is an important world public health problem with high morbidity and mortality. It is necessary to identify SCM biomarkers at the genetic level to identify new therapeutic targets and strategies.

**Method:** DEGs in SCM were identified by comprehensive bioinformatics analysis of microarray datasets (GSE53007 and GSE79962) downloaded from the GEO database. Subsequently, bioinformatics analysis was used to conduct an in-depth exploration of DEGs, including GO and KEGG pathway enrichment analysis, PPI network construction, and key gene identification. The top ten Hub genes were identified, and then the SCM model was constructed by treating HL-1 cells and AC16 cells with LPS, and these top ten Hub genes were examined using qPCR.

**Result:** STAT3, SOCS3, CCL2, IL1R2, JUNB, S100A9, OSMR, ZFP36, and HAMP were significantly elevated in the established SCM cells model.

**Conclusion:** After bioinformatics analysis and experimental verification, it was demonstrated that STAT3, SOCS3, CCL2, IL1R2, JUNB, S100A9, OSMR, ZFP36, and HAMP might play important roles in SCM.

## Introduction

Sepsis is an important world public health problem with high morbidity and remains one of the leading causes of death globally (Fleischmann et al., 2016). Sepsis is a complex disease that can cause acute multiple organ dysfunction and increased mortality. In 2016, sepsis and septic shock (sepsis-3) defined sepsis as life-threatening organ dysfunction resulting from a dysregulated host response to infection (Singer et al., 2016). The heart is one of the most vulnerable organs; the prevalence of sepsis patients complicated with myocardial injury is about 13.8%–40%, and the mortality rate is as high as 70%–90%, which seriously endangers human life and health (Lin et al., 2020). Septic cardiomyopathy (SCM), first described more than 40 years ago (Parker et al., 1984), is an acute cardiac disease caused by sepsis that is reversible and can recover in the early stages of sepsis (Beesley et al., 2018). The current recommended treatment strategy for SCM is symptomatic treatment, and there is no specific treatment for SCM patients (Lin et al., 2020). There is a need for a better understanding of the pathogenesis of SCM to identify new therapeutic targets and strategies. Therefore, identifying the key genes of SCM and finding effective biomarkers is crucial for the early diagnosis, prevention, and intervention of SCM.

Using a high-throughput gene expression analysis platform as a gene expression analysis tool to identify differentially expressed genes (DEGs), molecular functions, and signaling pathways involved in the occurrence and progression of diseases is a popular method in recent years (Canales et al., 2006). Gene Expression Omnibus (GEO, http://www.ncbi.nlm.nih.gov/geo/) is a public database containing a large number of gene expression profiles of various diseases (Edgar et al., 2002). This database provides an important resource for gene expression, functional analysis, and searching for DEGs of SCM. Previous studies have identified several biomarkers for SCM, such as STAT3, CCL2, MYC, and SERPINE1 (Chen et al., 2020a; Lin et al., 2020). However, these studies did not carry out experimental verification and only stayed on the results of bioinformatics analysis. This study conducted the first study on the cross-species screening of DEGs for SCM. In this study, bioinformatics analysis was used to find out the biological information hidden under the genome-wide expression microarray data and to screen out the DEGs in common between humans and mice with SCM. Then qPCR experiments were performed to validate the DEGs in order to provide a new diagnostic strategy for the screening of new biomarkers for SCM.

## Material and methods

### Data sources

Two gene expression profiles (GSE53007 and GSE79962) of SCM diseases were downloaded from the GEO database (http://www.ncbi.nih.gov/geo). The chip platform for GSE53007 gene expression profiling is GPL6885 (Illumina MouseRef-8 v2.0 expression beadchip), which consists of mouse heart tissue samples. The chip platform for GSE79962 gene expression profiling is GPL6244 (Affymetrix Human Gene 1.0 ST Array), which consists of human heart tissue samples. All data are publicly available for download online, and the study did not involve any experiments on humans or animals by any of the authors.

### Identification of differentially expressed genes

GSE53007 and GSE79962 were downloaded from the GEO database by using the GEOquery package of R software (version 4.2.1). Probes were converted to gene symbols according to the platform annotation information of the normalized data. Probes with more than one gene were eliminated, and the average of genes corresponding to more than one probe was calculated. The data were then normalized by the normalizeBetweenArrays function of the limma package (Ritchie et al., 2015). After data preprocessing, DEGs were analyzed using the limma package of R software (version 4.2.1). “*p* < 0.05 and Log (Fold Change) > 1 or Log (Fold Change) < −1” was defined as the threshold for screening DEGs. The data for the listed DEGs were processed, and heatmaps and volcano plots were drawn using the ComplexHeatmap and ggplot2 R packages, respectively.

### Enrichment analysis

In order to explore the pathways related to common differential genes between humans and mice, the DEGs of GSE53007 and GSE79962 were compared, the overlapping DEGs were identified, and then the pathway analysis of these DEGs was performed. Potential functional annotation in the Feature Enrichment Analysis Tool is based on the analysis of DEGs. Gene Ontology (GO) is a widely used tool for annotating genes with functions. The Kyoto Encyclopedia of Genes and Genomes (KEGG) enrichment analysis is a practical resource for the analysis of gene function and related high-level genomic functional information. The Database for Annotation, Visualization, and Integrated Discovery (DAVID; http://david.ncifcrf.gov) (version 6.8) was used to provide a comprehensive set of gene and protein functional annotation information (Huang et al., 2007). DAVID provides a comprehensive and systematic annotation of gene biology functions. After that, visualization was performed using the ggplot2 R package of R software (version 4.2.1). *p* < 0.05 was considered statistically significant.

### Protein–protein interaction network construction and hub gene identification

Protein–protein interaction (PPI) networks were generated by searching the Interacting Genes (STRING) database (https://string-db.org) using a search tool (Franceschini et al., 2013). A composite score >0.4 was considered statistically significant. Open-source bioinformatics software platform Cytoscape (version 3.8.2) was used to visualize molecular interaction networks (Smoot et al., 2011). Cytoscape software was used to analyze and visualize the biological network and nodal degree of DEGs. Based on MCC calculations, hub genes were identified using the Cytoscape plug-in software “cytoHubba.”

### Cell culture and treatment

Used lipopolysaccharide (LPS, from *E. coli* O111:B4, Sigma) treated mouse cardiomyocytes HL-1 cells and human cardiomyocyte AC16 cells to establish an SCM model to validate DEGs. HL-1 and AC16 were purchased from iCell Bioscience Inc. (Shanghai). HL-1 cells were cultured in MEM supplemented with 10% fetal bovine serum (FBS) and 1% penicillin/streptomycin. Culture conditions were as follows: gas phase: air, 95%; carbon dioxide, 5%; temperature, 37°C; incubator humidity, 70%–80%; and medium change every 2–3 days. HL-1 cells grew to about 80%, and the SCM model was established by treating with LPS 1 ug/ml for 24 h (Li et al., 2020a; Samokhvalov et al., 2015). AC16 cells were cultured in DMEM supplemented with 10% fetal bovine serum (FBS) and 1% penicillin/streptomycin, and the culture environment was the same as that of HL-1. AC16 cells established the SCM model according to the method of Pan et al. (2018) and were also treated with LPS 1 ug/ml for 24 h.

### Quantitative real-time PCR

The top ten genes identified by bioinformatics tools were individually validated in the SCM model established by LPS-treated HL-1 cells and AC16 cells. Extraction FastPure Cell/Tissue Total RNA Isolation Kit Cell/Tissue Total RNA Isolation Kit was used to extract RNA. First-strand cDNA was synthesized using the TaKaRa PrimeScript™ RT reagent Kit with gDNA Eraser (perfect real-time) (Code No. RR047A). Quantitative real-time PCR (qPCR) experiments were performed on an Applied Biosystems 7900HT Real-Time PCR System using TaKaRa TB Green® Premix Ex Taq™ II (Tli RNaseH Plus) (Code No. RR820A). The relative mRNA expression levels were calculated using the 2-ΔΔCT method. PCR reactions were normalized to the Actin. The primers were designed and synthesized by Sangon Biotech Co., Ltd. (Shanghai, China). The primer sequences are shown in Table 1. Two (STAT3 and CCL2) of the four hub genes obtained in the study by Chen et al., 2020b overlap with hub genes obtained in this study. The other two hub genes (MYC and SERPINE1), although not obtained in this study, were also tested at the transcriptional level (primer sequences are shown in Supplementary Table S1).

**TABLE 1:** Primers of top ten hub genes.

Gene names	Primer sequence (5′→3′)	
	Forward primer	Reverse primer
(A) Mouse		
STAT3	GCC​AAA​TGC​TTG​GGC​ATC​AA	AGG​TTC​CAA​TTG​GCG​GCT​TA
SOCS3	GCG​TAC​TGG​CCG​GGT​AAA​TA	GGA​GAG​ACA​GCG​GTC​GTA​AG
CCL2	GGC​TCA​GCC​AGA​TGC​AGT​TA	GCT​GCT​GGT​GAT​CCT​CTT​GT
IL1R2	AAG​GAA​CAA​CCA​CGG​AAC​CC	TGT​TAG​CCA​ACC​ACC​ACA​CA
TIMP1	CCA​GAA​CCG​CAG​TGA​AGA​GT	GTA​CGC​CAG​GGA​ACC​AAG​AA
JUNB	CAA​CCT​GGC​GGA​TCC​CTA​TC	GCC​TGT​GTC​TGA​TCC​CTG​AC
S100A9	AGG​AAG​GAA​GGA​CAC​CCT​GA	TGT​GTC​CAG​GTC​CTC​CAT​GA
OSMR	GCA​AGT​GCC​AAC​CAC​TTC​TG	CTC​CGA​CCA​CAC​TTG​TCT​CC
ZFP36	CCA​AGT​GCC​AGT​TTG​CTC​AC	ACT​TGT​GGC​AGA​GTT​CCG​TT
HAMP	GAA​AGC​AGG​GCA​GAC​ATT​GC	TCA​GGA​TGT​GGC​TCT​AGG​CT
Actin	TGT​TAC​CAA​CTG​GGA​CGA​CA	CTG​GGT​CAT​CTT​TTC​ACG​GT
(B) Human		
STAT3	AGT​GAC​CAG​GCA​GAA​GAT​GC	CAC​GTA​CTC​CAT​CGC​TGA​CA
SOCS3	ACC​TCA​GGC​TCC​TGG​TAG​AG	CCA​TGG​GAC​AGG​GAG​CAT​TT
CCL2	TCT​GTG​CCT​GCT​GCT​CAT​AG	TCT​TTG​GGA​CAC​TTG​CTG​CT
IL1R2	GAC​TCT​GGC​ACC​TAC​GTC​TG	TGA​ACG​GCA​GGA​AAG​CAT​CT
TIMP1	GGC​ATC​CTG​TTG​TTG​CTG​TG	GAA​CTT​GGC​CCT​GAT​GAC​GA
JUNB	CGA​CCA​CCA​TCA​GCT​ACC​TC	GTC​TGC​GGT​TCC​TCC​TTG​AA
S100A9	CAC​CCA​GAC​ACC​CTG​AAC​C	TGT​GTC​CAG​GTC​CTC​CAT​GA
OSMR	TAC​GCG​TCA​GAG​TTT​GCA​CT	TCC​ACT​TCA​CAG​TGG​TGC​TG
ZFP36	CAC​CTC​TTC​CCT​GCC​CAA​AT	ACC​AGG​AGA​CAC​TGG​AAC​CT
HAMP	CCA​CAA​CAG​ACG​GGA​CAA​CT	GCA​GCA​CAT​CCC​ACA​CTT​TG
Actin	ACAGAGCCTCGCCTTTGC	GCGGCGATATCATCATCC

### Statistical analysis

Statistical software SPSS 23.0 was used to perform a *t*-test on the changes in qPCR results, and *p* < 0.05 was considered statistically significant.

## Results

### Identification of differentially expressed genes in septic cardiomyopathy

**FIGURE 1:**
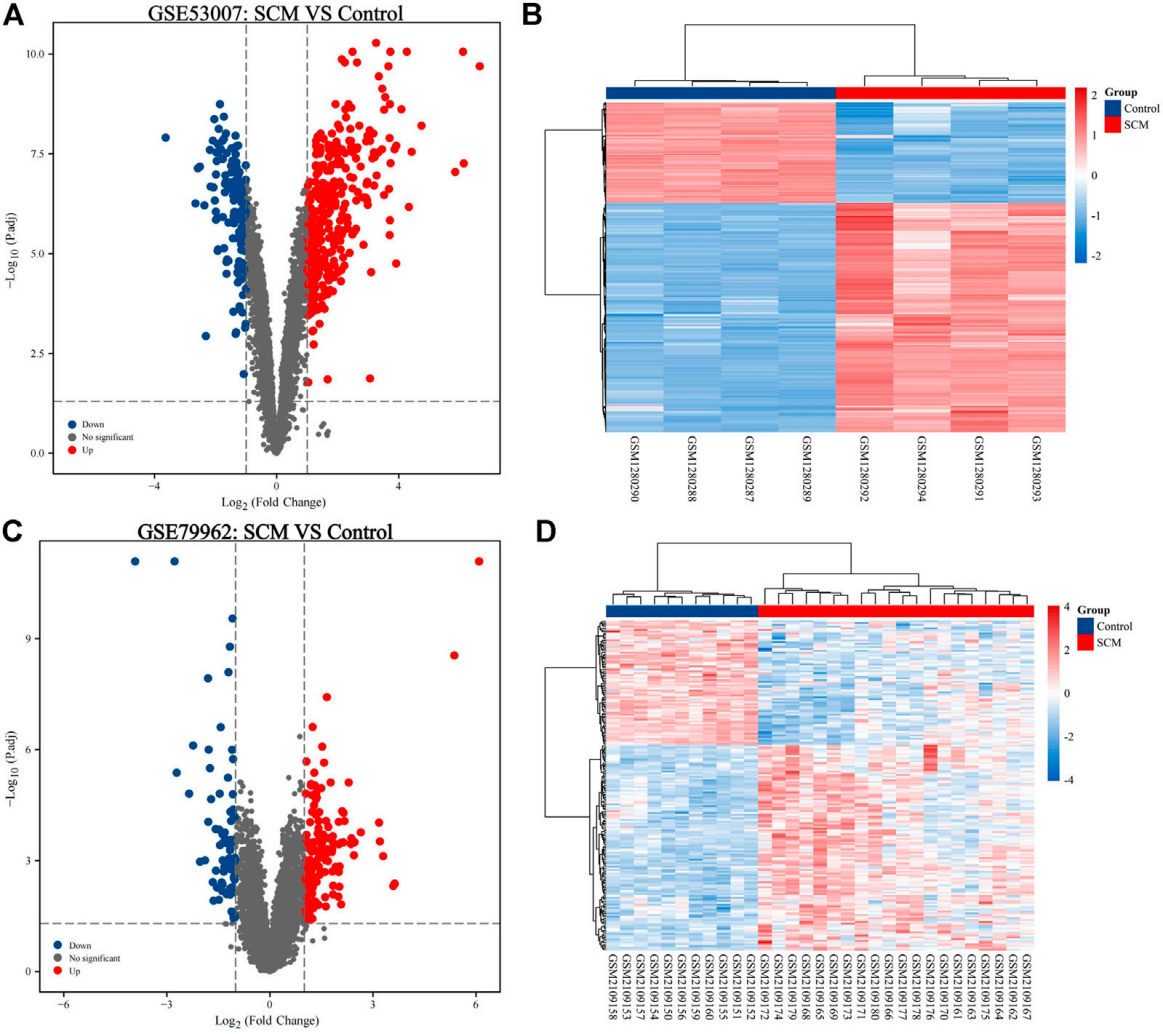
Analysis of DEGs. **(A)** Volcano map of DEGs based on GSE53007 (|logFC|>1, p. adj<0.05). **(B)** Heatmap of DEGs for GSE53007. **(C)** Volcano map of DEGs based on GSE79962 (|logFC|>1, p. adj<0.05). **(D)** Heatmap of DEGs for GSE79962.

**FIGURE 2:**
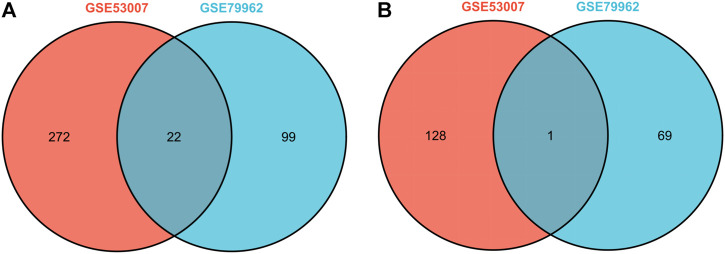
Venn diagram of overlapping upregulated DEGs of GSE53007 and GSE79962. **(A)** Two datasets overlapping upregulated DEGs. **(B)** Two datasets overlapping downregulated DEGs.

In this study, two SCM gene expression profiles, GSE53007 and GSE79962, were screened from the GEO database. Among them, GSE53007 was derived from mice and consisted of four normal mouse heart tissue samples and four SCM mouse heart tissue samples. GSE79962 was derived from humans and included 11 normal human heart tissue samples and 20 SCM patient heart tissue samples. Identified DEGs based on “*p* < 0.05 and |logFC|>1.” A total of 423 DEGs were identified from GSE53007, including 294 upregulated genes and 129 downregulated genes. A total of 121 upregulated genes and 70 downregulated genes were identified from GSE79962. Volcano plots for all genes and expression heatmaps for DEGs are shown in Figure 1. There were 22 upregulated genes and 1 downregulated gene overlapping in two datasets, plotted as a Venn diagram (Figure 2). The overlapping gene names are shown in Table 2.

**TABLE 2:** Screening of human and mouse common DEGs in SCM by comprehensive microarray analysis.

DEGs	Gene names
Upregulated	PTX3, S100A9, SOCS3, S100A8, TIMP1, HAMP, CYP1B1, OSMR, PNP, CCL11, ZFP36, PDK4, JUNB, CCL2, MID1IP1, TMEM2, STAT3, CP, SAT1, TGM2, IL1R2, and SLC7A5
Downregulated	ASB2

DEGs, differentially expressed genes; SCM, sepsis cardiomyopathy.

### Gene Ontology and Kyoto Encyclopedia of Genes and Genomes pathway analysis of differentially expressed genes

**FIGURE 3:**
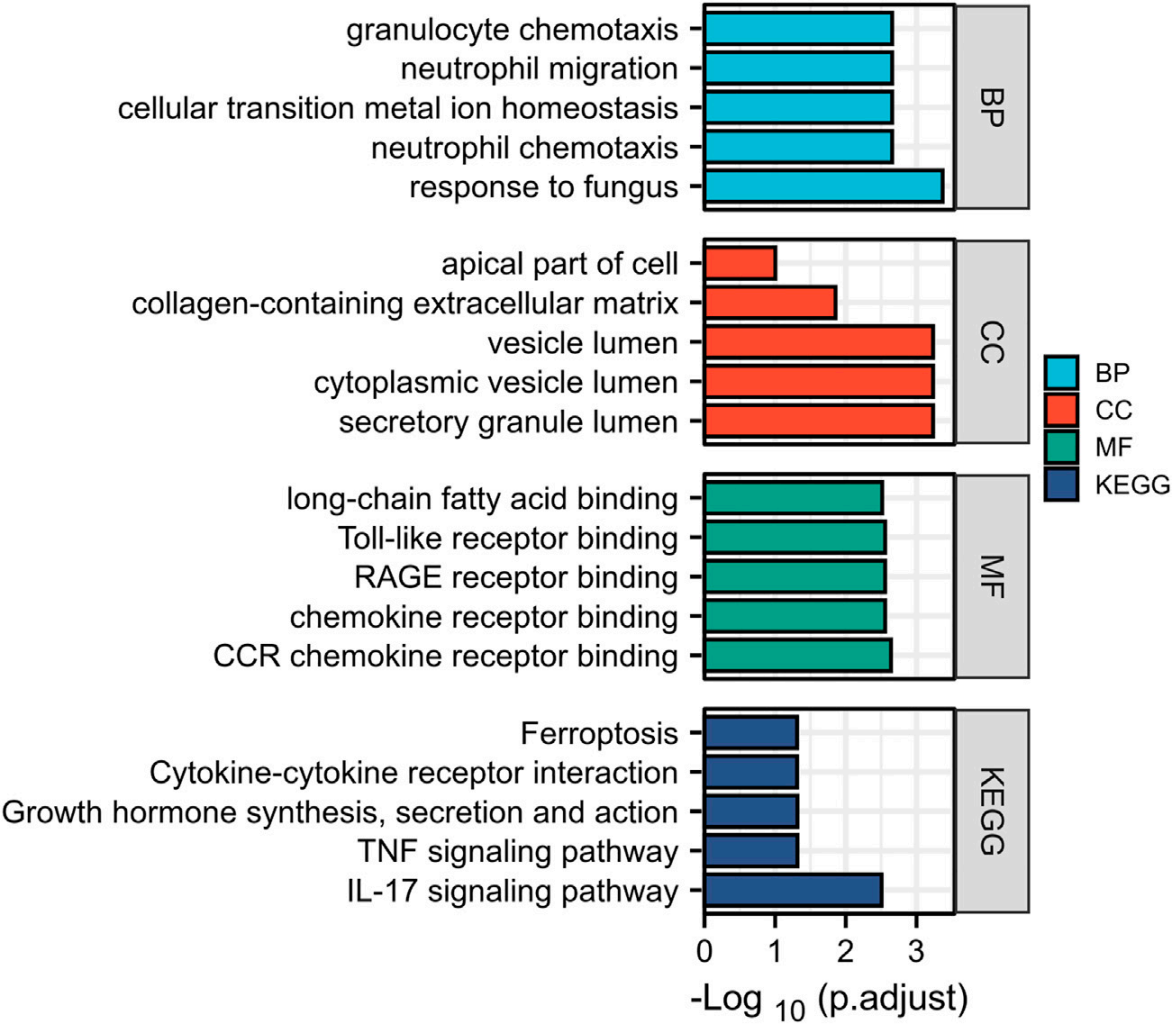
GO enrichment analysis and the KEGG pathway enrichment analysis of DEGs.

The DAVID online tool divides the GO enrichment analysis DEGs into three groups: BP, CC, and MF. As shown in Table 3 and Figure 3, in the biological processes group, the DEGs are mainly enriched in response to fungus, neutrophil chemotaxis, cellular transition metal ion homeostasis, neutrophil migration, and granulocyte chemotaxis. In the cellular component group, the DEGs are mainly enriched in secretory granule lumen, cytoplasmic vesicle lumen, vesicle lumen, collagen-containing extracellular matrix, and apical part of the cell. In the molecular function group, the DEGs are mainly enriched in CCR chemokine receptor binding, chemokine receptor binding, RAGE receptor binding, toll-like receptor binding, and long-chain fatty acid–binding. KEGG pathway enrichment analysis was performed on DEGs. Table 3 and Figure 3 show the KEGG pathway enrichment analysis of DEGs. These DEGs are mainly enriched in the IL-17 signaling pathway, TNF signaling pathway, growth hormone synthesis, secretion, and action, cytokine–cytokine receptor interaction, and ferroptosis.

**TABLE 3:** Significantly enriched GO terms and KEGG pathways of DEGs.

Category	Term	Description	Count	*p*-value
BP term	GO:0009620	Response to fungus	4	3.77071E-07
BP term	GO:0030593	Neutrophil chemotaxis	4	6.15222E-06
BP term	GO:0046916	Cellular transition metal ion homeostasis	4	7.14825E-06
BP term	GO:1990266	Neutrophil migration	4	1.0157E-05
BP term	GO:0071621	Granulocyte chemotaxis	4	1.197E-05
CC term	GO:0034774	Secretory granule lumen	5	2.32514E-05
CC term	GO:0060205	Cytoplasmic vesicle lumen	5	2.97748E-05
CC term	GO:0031983	Vesicle lumen	5	3.01986E-05
CC term	GO:0062023	Collagen-containing extracellular matrix	4	0.000965,599
CC term	GO:0045177	Apical part of cell	3	0.008574821
MF term	GO:0048020	CCR chemokine receptor binding	3	1.99252E-05
MF term	GO:0042379	Chemokine receptor binding	3	7.25262E-05
MF term	GO:0050786	RAGE receptor binding	2	8.05906E-05
MF term	GO:0035325	Toll-like receptor binding	2	9.66358E-05
MF term	GO:0036041	Long-chain fatty acid binding	2	0.00013304
KEGG pathway	hsa04657	IL-17 signaling pathway	4	4.64858E-05
KEGG pathway	hsa04668	TNF signaling pathway	3	0.001820407
KEGG pathway	hsa04935	Growth hormone synthesis, secretion, and action	3	0.002165753
KEGG pathway	hsa04060	Cytokine-cytokine receptor interaction	4	0.003557286
KEGG pathway	hsa04216	Ferroptosis	2	0.003654574

DEGs, differentially expressed genes; GO, Gene Ontology; BP, biological processes; CC, cellular component; MF, molecular function; KEGG, the Kyoto Encyclopedia of Genes and Genomes.

### Protein–protein interaction network analysis of differentially expressed genes

**FIGURE 4:**
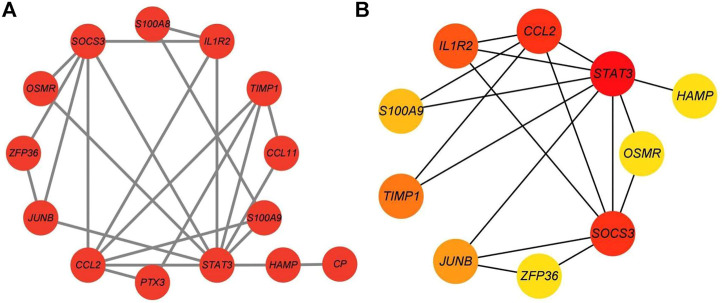
PPI network of DEGs constructed using Cytoscape. **(A)** PPI network containing 23 nodes and 24 edges constructed based on the STRING online database and visualized using Cytoscape. **(B)** The most significant genes obtained from the PPI network.

A PPI network was created using the STRING database to further explore the biological properties of these DEGs. The PPI network with 23 nodes and 24 edges was constructed based on the STRING online database and visualized by Cytoscape software (Figure 4A). The top ten hub genes based on MCC calculations were identified using the Cytoscape plug-in software “cytoHubba” (Figure 4B). The top ten hub genes are STAT3, SOCS3, CCL2, IL1R2, TIMP1, JUNB, S100A9, OSMR, ZFP36, and HAMP.

### Quantitative real-time PCR of differentially expressed genes

**FIGURE 5:**
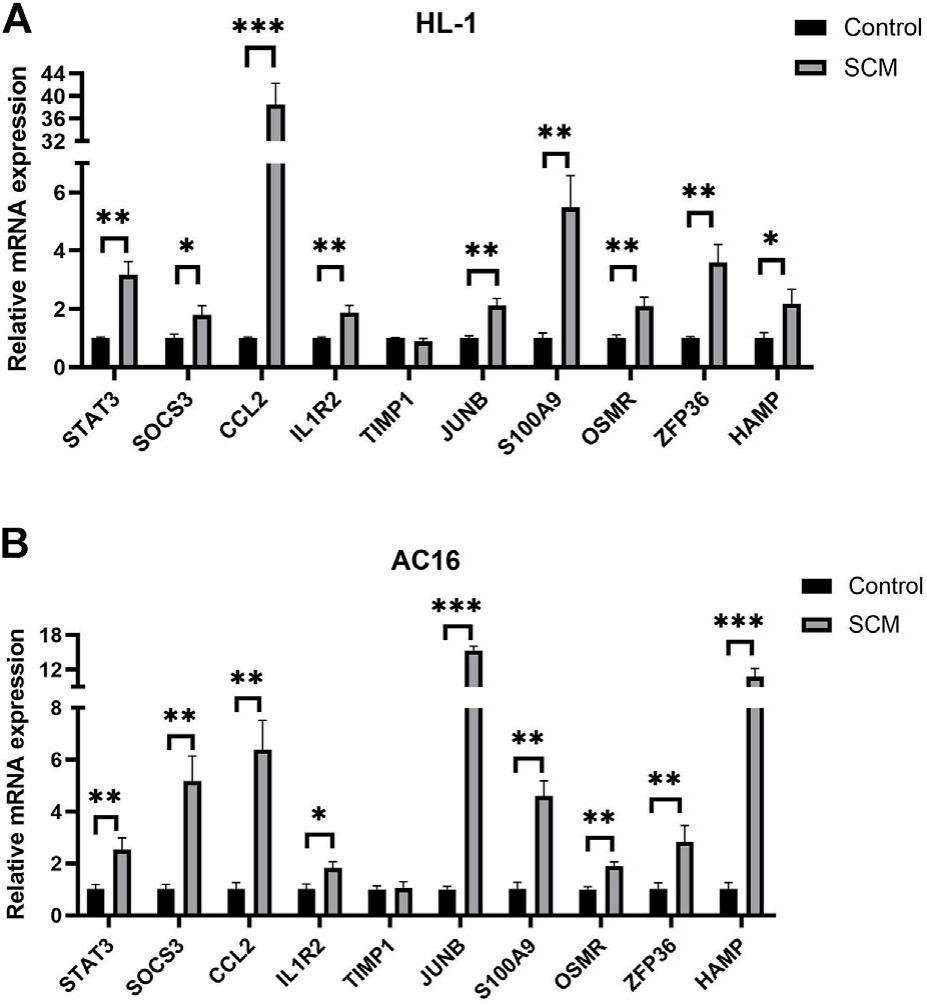
Results of Quantitative real-time PCR experiments for the top ten genes (*<0.05, **<0.01, ***<0.001). **(A)** Expression of Hub gene in HL-1 cells. **(B)** Expression of Hub gene in AC16 cells.

Quantitative real-time PCR (qPCR) was used to detect and compare the expression of the identified genes (Figure 5). Nine hub genes were significantly elevated in the LPS-treated HL-1 cell SCM model, including STAT3, SOCS3, CCL2, IL1R2, JUNB, S100A9, OSMR, ZFP36, and HAMP (Figure 5A). The same results were obtained in the SCM model constructed by LPS-treated AC16 cells (Figure 5B). Transcript levels of both MYC and SERPINE1 were elevated in both HL-1 cells and AC16 cells after LPS-treated (Supplementary Figure S1).

## Discussion

Sepsis, defined as a life-threatening organ dysfunction due to a dysregulated immune response to infection (Singer et al., 2016), has become one of the top ten causes of death in both developed and developing countries (Hawiger, 2018), with mortality rates as high as 30% (Morin et al., 2015). SCM is an acute cardiac disease caused by sepsis (Beesley et al., 2018), which is reversible and can be recovered in the early stages of sepsis (Lu et al., 2020). Although SCM is reversible in the early stage, its pathogenesis is still unclear, and the mortality rate is high (Lin et al., 2020). Therefore, it is crucial for the early identification of SCM. Microvascular damage caused by “Genomic Storm” has been shown to be the pathogenesis of sepsis (Hawiger, 2018). Gene therapy for SCM is also constantly being explored. Ma et al. (2016) found that increased myocardial miR-125b expression attenuated sepsis-induced cardiac insufficiency and improved survival, suggesting that miR-125b may be a target of SCM. Zhang et al. (2020) showed that HSPA12B prevents sepsis-induced severe cardiomyopathy by regulating the expression of miR-126 targeting adhesion molecules, thereby reducing the accumulation of immune cells in the myocardium. Other research has shown that miR-21-3p controls sepsis-related cardiac dysfunction by regulating SORBS2, suggesting that miR-21-3p may be a potential target for SCM therapy (Wang et al., 2016). It can be seen that gene therapy will be the first choice for the treatment of SCM in the future.

In this study, a total of 23 DEGs (22 upregulated genes and 1 downregulated gene) were identified in SCM by comprehensive bioinformatics analysis of microarray datasets (GSE53007 and GSE79962). Subsequently, bioinformatics analysis was used to conduct an in-depth exploration of DEGs, including GO and KEGG pathway enrichment analysis, PPI network construction, and key gene identification. In this study, the top ten Hub genes were identified, and then the SCM model was constructed by treating HL-1 cells and AC16 cells with LPS, and these top ten Hub genes were examined using qPCR. After the experiment, it was found that the expressions of STAT3, SOCS3, CCL2, IL1R2, JUNB, S100A9, OSMR, ZFP36, and HAMP were significantly increased in HL-1 cells and AC16 cells treated with LPS. This suggests that these genes may play an important role in SCM.

Signal transducer and activator of transcription 3 (STAT3) are important for biological functions in embryogenesis, immunity, hematopoiesis, and cell migration (Liu et al., 2021). STAT3 is a functionally diverse molecule that acts as a master signaling transcription factor in immune and inflammatory pathways, and its overactivation or inactivation can lead to human disease, suggesting that STAT3 function is critical to human health (Hillmer et al., 2016). Studies have shown a critical role of STAT3 in the pathophysiology of sepsis (Hilliard et al., 2015; Huang et al., 2018). Kano et al. (2003) found that mice with conditional knockout of the STAT3 gene in macrophages and neutrophils were highly susceptible to sepsis, resulting in excessive systemic inflammation and higher mortality. However, Lee et al. (2012) found that inhibition of STAT3 activity ameliorated organ inflammatory responses in the LPS-induced sepsis model. These may demonstrate that STAT3 has multiple effects during septic injury. Studies have found that the reduction of STAT3 levels in the myocardium is involved in the mechanism of mesenchymal stem cell exosomal miR-223-induced reduction of inflammation and cell death in the cecal ligation and puncture mice (Wang et al., 2015). Changes in STAT3 activity or expression are involved in the pathological process of SCM.

SOCS family proteins are part of the classical negative feedback system that regulates cytokine signaling. Suppressor of cytokine signaling 3 (SOCS3) is involved in the negative regulation of cytokine signaling through the JAK/STAT pathway and inhibits cytokine signaling by binding to tyrosine kinase receptors (Gao et al., 2018). Shangxun et al. (2020) found that ADAR1 is expected to reduce IL-6 levels by regulating the expression of SOCS3, thereby inhibiting inflammation and reducing sepsis. SOCS3 is also linked to STAT3, and more and more reports have shown that SOCS3 and STAT3 are abnormally expressed in different bone marrow and lymphocytes and various non-hematopoietic cells, suggesting their involvement in various infections and inflammatory diseases (Gao et al., 2018). The role of SOCS3 in SCM still needs further study.

C–C motif chemokine 2 (CCL2) is a chemokine that attracts monocytes and basophils but not neutrophils or eosinophils. In recent years, the role of the chemokine CCL2 and its major receptor CCR2 in cancer pathogenesis has received special attention, and elevated levels of CCL2 have been associated with increased growth and progression of a variety of cancers (Hao et al., 2020). The CCL2–CCR2 signaling axis has been implicated in many inflammatory and neurodegenerative diseases, such as atherosclerosis, multiple sclerosis, asthma, neuropathic pain, and diabetic nephropathy (O’Connor et al., 2015); therefore, it has also been explored as a potential target for the treatment of these diseases. Yucel et al. (2017) reported increased expression levels of CCL2 in an LPS-induced inflammation model using human-induced pluripotent stem cell–derived cardiomyocytes. CCL2 may be a potential target for SCM therapy.

Interleukin-1 receptor type 2 (IL1R2) is a decoy receptor of the interleukin 1 (IL1) receptor family responsible for capturing IL1 and reducing IL1 bioavailability (Peters et al., 2013). IL1R2 is elevated in sepsis and correlates with disease severity (Giri et al., 1994; Muller et al., 2002). Lang et al. (2017) discovered for the first time that serum IL1R2 is a biomarker for the diagnosis and identification of *Escherichia coli*, *Staphylococcus aureus*, and G+/G− bacterial sepsis, which is more sensitive and specific than traditional biomarkers such as PCT and CRP.

Transcription factor jun-B (JUNB) is a proto-oncogene. JUNB is a cell proliferation inhibitor, senescence inducer, and tumor suppressor (Piechaczyk and Farras, 2008). The expression of JUNB is increased in a variety of cancers, and studies have shown that JUNB plays an important role in cancer metastasis (Kallergi et al., 2019). Konishi et al. (2008) found that JUNB is an important upstream regulator of p16, which helps maintain cell senescence, thereby preventing the malignant transformation of Transient amplifying cells, and plays an important role in controlling the occurrence of prostate cancer. In this study, the expression of JUNB was increased in SCM, but its specific function needs to be further explored.

S100A9 is a calcium- and zinc-binding protein that plays an important role in regulating inflammatory processes and immune responses. S100A9 is a pro-inflammatory alarmin that is upregulated in inflamed tissues (Ding et al., 2021). Numerous studies have shown that S100A9 is upregulated in various inflammatory and infectious diseases, including sepsis and patients with severe COVID-19 infection (Chen et al., 2020a; Dubois et al., 2019; Holzinger et al., 2019; Liao et al., 2021; Marinkovic et al., 2020; Wang et al., 2014). The findings of Alkhateeb et al. (2019) suggest that genetic targeting of S100A9 in mice suppresses the expansion of myeloid-derived suppressor cells during advanced sepsis, which may have biological implications. S100A9 may be a potential therapeutic target based on the finding in this study that S100A9 is upregulated in SCM.

Oncostatin-M-specific receptor subunit beta (OSMR), a member of the interleukin 6 (IL6) receptor family, functions as a major mediator of cardiomyocyte remodeling under pathological conditions (Kubin et al., 2022). The involvement of OSMR in multiple human heart diseases such as aortic stenosis, myocardial infarction, myocarditis, and various cardiomyopathy makes OSMR a promising new therapeutic target (Kubin et al., 2022). Wu et al. (2021) found that long noncoding RNA Pvt1 regulates pathological cardiac hypertrophy through miR-196b-mediated OSMR regulation. The expression of OSMR is upregulated in SCM, and it is crucial to study its role in SCM.

Zinc finger protein 36 (ZFP36) family proteins are RNA-binding proteins involved in messenger RNA (mRNA) metabolic pathways. ZFP36 plays an important role in regulating immune responses and inflammatory diseases by inhibiting the production of various inflammatory cytokines such as TNF-alpha in macrophages (Lai et al., 1999). Zhang et al. (2013) found that ZFP36 is expressed in vascular endothelial cells and macrophage foam cells and inhibits the expression of pro-inflammatory mRNA transcripts, and enhanced vascular ZFP36 expression may reduce vascular inflammation. ZFP36 may be a novel target for the treatment of SCM.

HAMP is a gene that regulates iron metabolism. Shen et al. (2019) showed that HAMP functions as a tumor suppressor gene, the role of HAMP in cell proliferation and metastasis is related to cell cycle checkpoints, and HAMP can be regarded as a diagnostic biomarker and targeted therapy for hepatocellular carcinoma. Scindia et al. (2019) found novel preventive and therapeutic roles for hepcidin in sepsis-related bacteremia, AKI, and mortality. In SCM, HAMP is a target worth investigating.

Chen et al. (2020b) performed bioinformatics analysis on human gene chips in a previous study and obtained four hub genes (STAT3, CCL2, MYC, and SERPINE1), but they have not been experimentally verified. This study also verified these four genes; in addition to the previously discussed STAT3 and CCL2, MYC and SERPINE1 are also highly expressed in the SCM cells model. Studies have shown that MYC may be involved in the process of LPS-induced sepsis by promoting cell proliferation and inhibiting apoptosis, and MYC may reduce inflammation during the progression of LPS-induced sepsis (Li et al., 2020b). SERPINE1 is also known as PAI-1. A study found that the first week of plasma PAI-1 level was associated with sepsis severity and mortality and could be used as a prognostic biomarker in sepsis (Lorente et al., 2014).

## Conclusion

In this study, the top ten key genes of SCM were screened out by comprehensive bioinformatics analysis of gene chips. After experimental verification, it was found that the differences in nine genes were statistically significant. This study may provide new ideas for early screening, diagnosis, and treatment of SCM. This study suggests that STAT3, SOCS3, CCL2, IL1R2, JUNB, S100A9, OSMR, ZFP36, and HAMP may play important roles in SCM. In follow-up further studies, it is necessary to clarify the association between these DEGs and SCM, explore their underlying mechanisms, and find their potential therapeutic targets.

## Data Availability

The original contributions presented in the study are included in the article/Supplementary Materials; further inquiries can be directed to the corresponding author.
