# Supplementary material for: Identification of novel biomarkers in septic cardiomyopathy via integrated bioinformatics analysis and experimental validation
Source: Front Genet. 2022 Jul 25;13:929293. doi: 10.3389/fgene.2022.929293 (PMC9358039; doi:10.3389/fgene.2022.929293)
Supplement: Supplementary file 1 [file DataSheet1.docx]

Supplementary Material

# Supplementary Tables

**Supplementary Table 1.** The primers of MYC and SERPINE1

| Gene names | | Primer sequence (5’→3’) | |
| --- | --- | --- | --- |
|  |  | Forward primer | Reverse primer |
| Mouse | MYC | TGTGGAGAAGAGGCAAACCC | TTGTGCTGGTGAGTGGAGAC |
|  | SERPINE1 | TTTGTCATCTCAGCCCGCAT | GCGTCTCTTCCCACTGTCAA |
| Human | MYC | GTCACACCCTTCTCCCTTCG | CGGGTCGCAGATGAAACTCT |
|  | SERPINE1 | CAGCTCATCAGCCACTGGAA | CATGTCGGTCATTCCCAGGT |

## Supplementary Figures


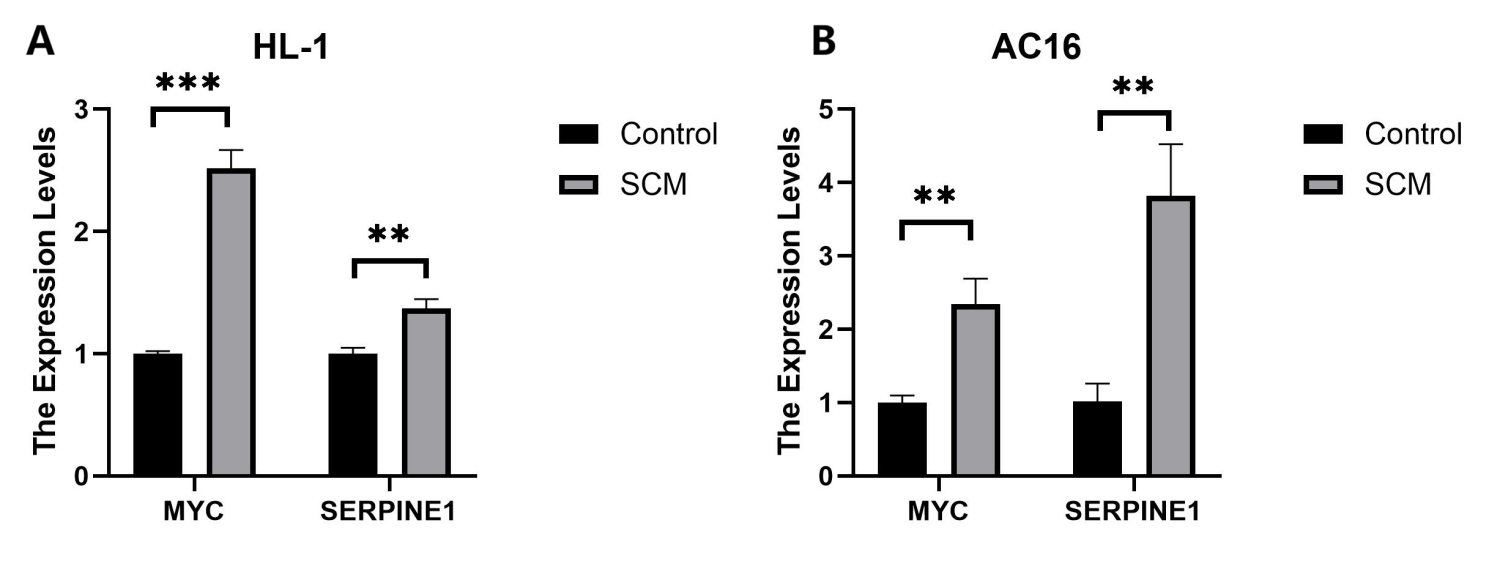


**Supplementary Figure 1.** Results of Quantitative real-time PCR experiments for MYC and SERPINE1 (**<0.01, ***<0.001).
